# Supplementary material for: Nuclear VANGL2 Inhibits Lactogenic Differentiation
Source: Cells. 2024 Jan 25;13(3):222. doi: 10.3390/cells13030222 (PMC10854645; doi:10.3390/cells13030222)
Supplement: Supplementary file 1 [file cells-13-00222-s001.zip › cells-2782878-supplementary.pdf]

SUPPLEMENTARY FIGURES

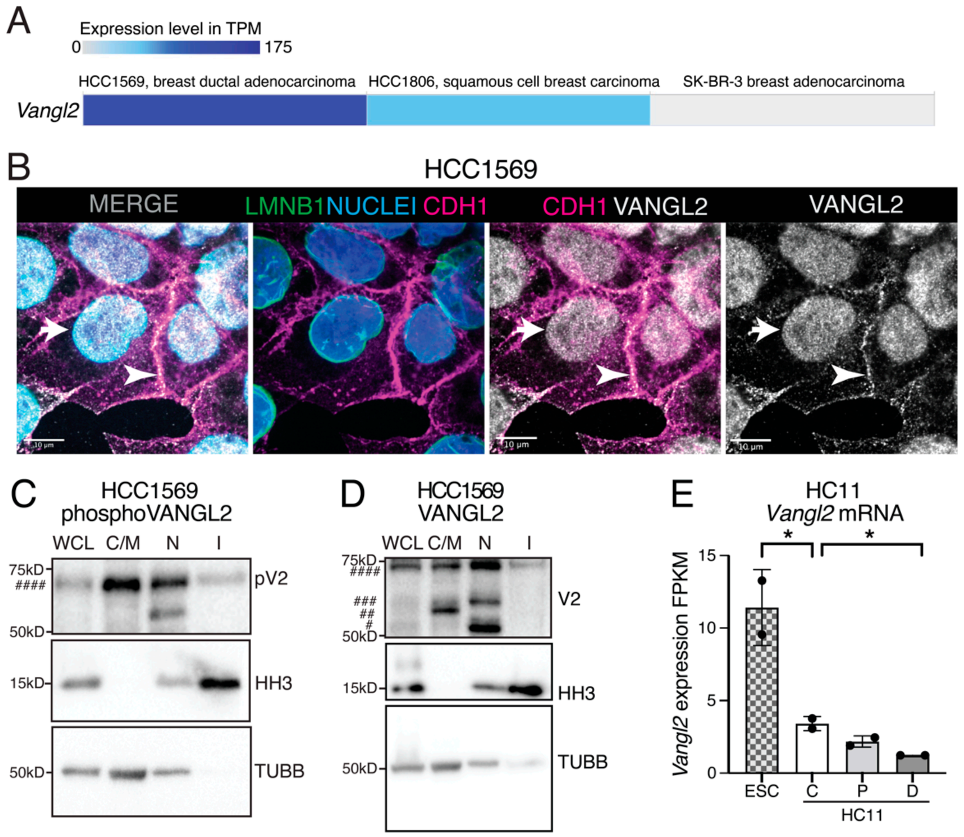

Supplementary Figure S1. VANGL2 localizes to the nucleus in HCC1569 cells.

(A) Relative Vangl2 expression in three breast cancer cell lines from ebi.ac.uk/gxa/home. HCC1569 was chosen for our study due to its relatively high level of *Vangl2*.

(B) Representative immunofluorescence photomicrographs of HCC1569 cells immunostained for Lamin B1 (LMNB1, green), E-cadherin (CDH1, pink) and VANGL2 (white, arrows indicate nuclear staining, arrowheads indicate plasma membrane staining), with nuclei labeled in Hoechst (blue).

(C) Representative Western blot of phosphoVANGL2 (pV2) from whole cell lysates (WCL) that were fractionated as follows: cytosolic/membrane (C/M), soluble nuclear (N), and insoluble nuclear (IN). Fractions were assessed using specific antibodies: ß-tubulin I (TUBB) for cytoplasmic/membrane fraction and histone H3 (HH3) for nuclear fraction. One form of VANGL2 is observed: hyperphosphorylated, ####.

(D) Representative Western blot of VANGL2 (V2) from whole cell lysates (WCL) that were fractionated and assessed using specific antibodies as described in (C). Four forms of VANGL2 are observed: unphosphorylated, #; hypophosphorylated, ##; phosphorylated, ###; and hyperphosphorylated, ####.

(E) RNA-seq data shown as fragments per kilobase per million reads (FPKM) for *Vangl2* in HC11 cells over the time course of differentiation: confluence (C), primed (P) and day 3 of differentiation (D), compared to *Vangl2* expression in embryonic stem cells (ESC).

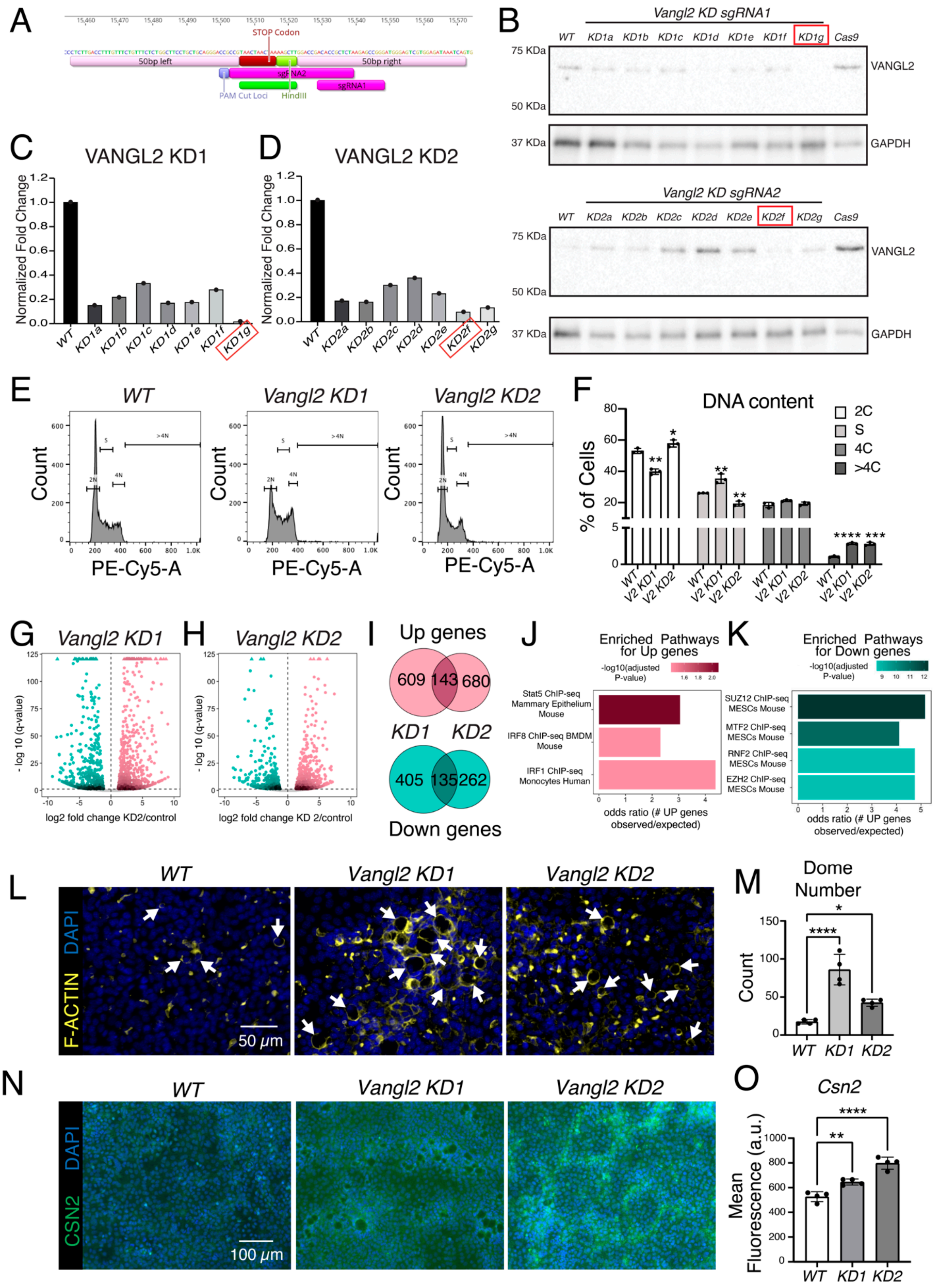
A
STOP Codon
50bp left
50bp right
sgRNA2
sgRNA1
PAM Cut Loci
HindIII
B
Vangl2 KD sgRNA1
WT KD1a KD1b KD1c KD1d KD1e KD1f KD1g Cas9
75 KDa
50 KDa
37 KDa
VANGL2
GAPDH
Vangl2 KD sgRNA2
WT KD2a KD2b KD2c KD2d KD2e KD2f KD2g Cas9
C
VANGL2 KD1
Normalized Fold Change
D
VANGL2 KD2
E
WT
Vangl2 KD1
Vangl2 KD2
Count
PE-Cy5-A
F
DNA content
% of Cells
2C
S
4C
>4C
WT V2 KD1 V2 KD2
G
Vangl2 KD1
- log 10 (q-value)
log2 fold change KD2/control
H
Vangl2 KD2
log2 fold change KD 2/control
I
Up genes
609 143 680
KD1 KD2
405 135 262
Down genes
J
Enriched Pathways for Up genes
-log10(adjusted P-value)
Stat5 ChIP-seq Mammary Epithelium Mouse
IRF8 ChIP-seq BMDM Mouse
IRF1 ChIP-seq Monocytes Human
odds ratio (# UP genes observed/expected)
K
Enriched Pathways for Down genes
-log10(adjusted P-value)
SUZ12 ChIP-seq MESCs Mouse
MTF2 ChIP-seq MESCs Mouse
RNF2 ChIP-seq MESCs Mouse
EZH2 ChIP-seq MESCs Mouse
odds ratio (# UP genes observed/expected)
L
WT
Vangl2 KD1
Vangl2 KD2
F-ACTIN DAPI
50 μm
M
Dome Number
Count
WT KD1 KD2
N
WT
Vangl2 KD1
Vangl2 KD2
CSN2 DAPI
100 μm
O
Csn2
Mean Fluorescence (a.u.)
WT KD1 KD2

Supplementary Figure S2. *Vangl2* KO cells express proteins and genes involved in lactogenic differentiation.

(A) Cartoon representation of CRISPR/Cas9 strategy to knockdown *Vangl2* using two different RNA guide sets.

(B-D) Western blots (B) and quantification of VANGL2 in *Vangl2 KD*1 (C) and *Vangl2 KD* 2 (D) HC11 cells. Red boxes indicate *KD1* and *KD2* HC11 cells selected for further study.

(E) Representative FACS histograms for DNA content analysis of undifferentiated *WT*, *Vangl2 KD1* and *Vangl2 KD2* HC11 cell lines as labeled by propidium iodide (PE-Cy5-A).

(F) Quantification of FACS DNA content analysis showing the percentage of undifferentiated *WT*, *Vangl2 KD1* and *Vangl2 KD2* HC11 cells with 2C, 4C or > 4C DNA content or in the S phase.

(G, H) Volcano plots showing the differential gene expression of the *Vangl2 KD1* and *Vangl2 KD2* cells in comparison to *WT*. Each dot represents a gene. Teal color shows genes with decreased expression and pink shows genes with increased expression.

(I) (Top) Venn diagram showing the number of upregulated (Up) genes in *Vangl2 KD1* (609) and in *Vangl2 KD2* (680). (Bottom) Venn diagram showing the number of downregulated (Down) genes in *Vangl2 KD1* (405) and *Vangl2 KD2* (262). The shared differentially expressed genes (Up: 143, Down: 135) were used to generate the enriched pathway plots.

(J, K) Enriched pathways from the genes upregulated in *Vangl2 KD1* cells (J) and downregulated in *Vangl2 KD2* cells (K). The colored scale is logarithmic, with, the darker colors representing higher p-values.

(L, M) Representative immunofluorescence photomicrographs of *WT*, *Vangl2 KD1* and *Vangl2 KD2* HC11 cells at differentiation day 2 immunostained for F-actin (yellow), with nuclei labeled with Hoechst (blue) (L), and quantification of the number of domes (arrows) (M).

(N, O) Representative photomicrographs of *WT*, *Vangl2 KD1* and *Vangl2 KD2* HC11 cells at differentiation day 2 immunostained for CSN2 (green), with nuclei labeled with Hoechst (blue) (N), and quantification of CSN2 mean fluorescence (O).

Error bars represent mean ± SD. Error bars represent mean ± SD. Analyzed by two-tailed unpaired Student's t-test. n=3 biological replicates. p values: * < 0.05, **< 0.01, *** < 0.001, **** < 0.0001.

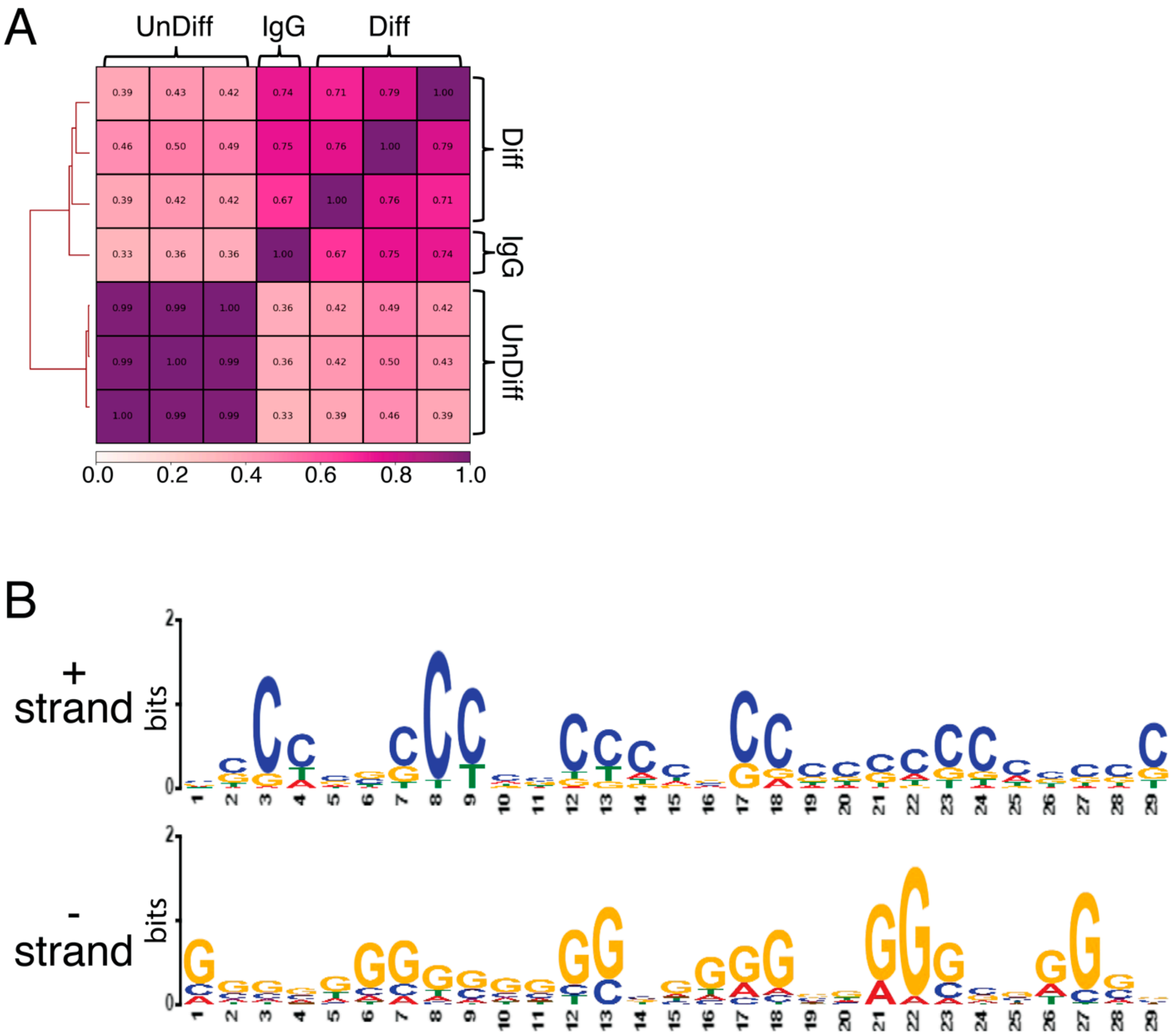

Supplementary Figure S3. VANGL2 binds DNA motifs in undifferentiated, but not differentiated, HC11 cells, including one in the *Stat5a* promoter.

(A) Heatmap of the Pearson correlation measure over the genome-wide signal for CUT&RUN reads of undifferentiated (unDiff) and differentiated (Diff) HC11 cells using a rabbit anti-VAGNLL2 antibody or non-specific rabbit IgG. Rows are clustered according to inter-sample correlation. Color intensity is proportional to the correlation value reported in each cell.

(B) Representation of the motif found in the *Stat5a* promoter (forward + and reverse – DNA strand).

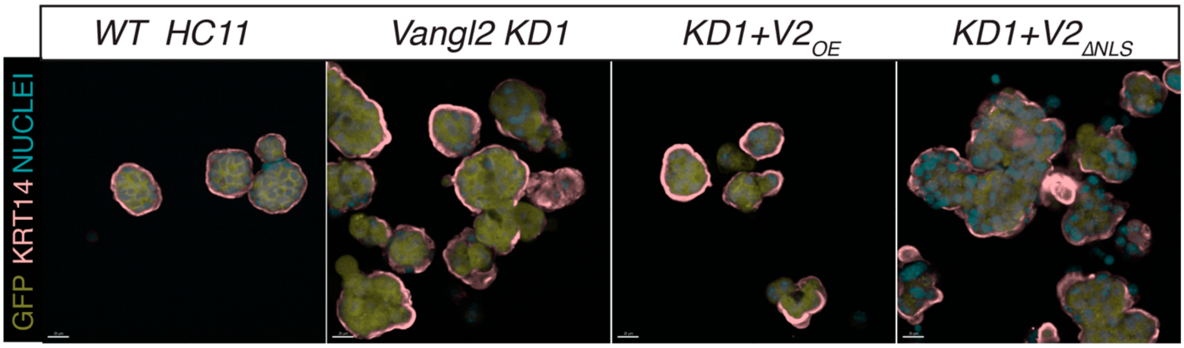

Supplementary Figure S4. Re-expression of *Vangl2* constructs in *Vangl2* KD cells.

(A) Representative immunofluorescence micrographs of acini grown in 3D Matrigel: *WT HC11*, *Vangl2 KD1*, and *Vangl2 KD1* HC11 cells overexpressing either *WT Vangl2* (V2$_{OE}$) or *Vangl2 DNLS* (V2$_{DNLS}$) lentiviral constructs. Acini are immunostained for KRT14 (pink) and GFP (yellow), with nuclei labeled with Hoechst (blue). GFP labeling shows lentiviral transduction.
